# Sex-dependent phenotypes of anxiety and palatable food-seeking in offspring born from rats exposed to a high-fat diet

**DOI:** 10.3389/fpsyg.2026.1898577

**Published:** 2026-08-26

**Authors:** Estefania Espitia-Bautista, Anel Cruz-Lemolle, Carlos Sánchez-Meza, Daniela Cortés-Gutiérrez, Haydée Lugo-Martínez, Carolina Escobar

**Affiliations:** 1 Instituto Nacional de Psiquiatría Ramón de la Fuente Muniz, Mexico City, Mexico; 2 Departamento de Anatomía, Facultad de Medicina, UNAM, Mexico City, Mexico; 3 Departamento de Embriología y Genética, Facultad de Medicina, UNAM, Mexico City, Mexico

**Keywords:** anxiety-like behavior, food-seeking behavior, high-fat diet, intergenerational effects, offspring

## Abstract

**Introduction:**

Fat-rich diet not only affects individuals who consume it, but it can also impact their offspring, leading to inherited metabolic disruption and potentially modifying behavior. Behavioral changes may favor a preference for palatable foods and the emergence of anxiety-like behaviors. This study aimed to evaluate the preference for palatable food, food-seeking, and anxiety behaviors in the offspring (F1) of parents that consumed a high-fat diet (F0).

**Methods:**

Wistar rats were maintained for 18 weeks after weaning in one of two dietary conditions: 1) chow (C) or 2) high-fat diet (HF). Diet-specific mated parents (F0) were used to obtain the offspring (F1), forming four groups based on parental diet: 1) both parents fed chow diet (C-Pa), 2) father fed HF diet and mother fed chow diet (HF-Fa), 3) mother fed HF diet and father fed chow diet (HF-Mo), and 4) both parents fed HF diet (HF-Pa). Offspring were only exposed to the regular chow diet. At 40 days of age, the offspring were exposed to a modified open field test to evaluate food-seeking and anxiety behaviors. Starting on PN41 all groups were exposed to 10% sucrose solution for 1 hr daily for 3 weeks and then were evaluated in the place preference light/dark box test to measure the motivation to consume sucrose water in the light compartment.

**Results:**

Male and female offspring (F1) from the HF-Pa group gained more body weight and consumed more chow; females HF-Pa exhibited fewer anxiety-like behaviors in the modified open field test and lower palatable food seeking behaviors, while male HF-Pa showed lower food seeking behavior for chow. After 3 weeks of sucrose administration HF-Pa males and females showed less motivation to consume sucrose water in the light/dark box test.

**Discussion:**

Offspring born from both parents consuming HF diet present the most significant metabolic and behavioral changes, with more pronounced effects in females; indicating that a fat rich diet when consumed by both parents is a risk factor for their offspring by affecting anxiety and palatable food-seeking behaviors.

## 1 Introduction

The consumption of fat-rich diets constitutes a relevant environmental factor driving metabolic and neurobehavioral health problems in modern society. Fat-rich diets act as potent orosensory stimuli that change the brain’s reward circuitry, activating mesolimbic dopaminergic pathways similar to those of substance of abuse (Gearhardt et al., 2011; Corwin and Babbs, 2012; Volkow et al., 2013). This activation overrides natural satiety signals and promotes compulsive overeating (Small and Difeliceantonio, 2019). Emerging evidence highlights that fat-rich diet consumption contributes to the dysregulation of energy homeostasis, impairment of insulin sensitivity, chronic low-grade inflammation, mitochondrial dysfunction, disruptions in lipid and glucose metabolism, and the onset of metabolic disorders such as obesity, type 2 diabetes, and non-alcoholic fatty liver disease (Hotamisligil, 2017; Popkin et al., 2017; Abbafati et al., 2020). The Developmental Origins of Health and Disease (DOHaD) paradigm establishes that parental environmental experiences, including nutrition, can program the physiology and behavior of offspring through epigenetic mechanisms (Barker, 2007; Waterland and Michels, 2007). Therefore, the nutritional state of parents can influence the future metabolic conditions of their offspring.

Animal models have extensively explored the intergenerational and transgenerational transmission of parental high-fat diet (HF) consumption on their offspring (Dunn and Bale, 2011; Fullston et al., 2013; Lane et al., 2015; Romaní-Pérez et al., 2017; Zhou et al., 2018; Escalona et al., 2023; Larqué et al., 2023). These studies indicate that the effects of hypercaloric diets on offspring (F1) can differ by sex. Camacho et al., 2017 reported that mother rats exposed to hypercaloric diets produced female offspring with exacerbated preference responses to palatable food as compared to males (Camacho et al., 2017). In a previous study, we reported that male offspring born from dams fed a HF developed elevated visceral adipose tissue, together with increased insulin and leptin levels when compared with male F1 offspring born from dams fed with a standard diet (Escalona et al., 2023; Larqué et al., 2023). Research shows that paternal HF consumption leads to metabolic disturbances in offspring, such as increased adiposity, insulin resistance, and impaired glucose metabolism (Ng et al., 2010; Fullston et al., 2012). Moreover, offspring exhibited epigenetic changes in specific genes, predisposing them to conditions like diabetes, obesity, and cardiovascular diseases (Dimofski et al., 2021), as well as some cognitive effects persisting into the third generation (Zhou et al., 2018).

While the intergenerational inheritance of metabolic dysregulation is documented, the effects on behavioral phenotypes involving motivated behavior for food are less clear. HF consumption overrides natural satiety signals and promotes compulsive overeating in F0 (Small and Difeliceantonio, 2019; Espitia-Bautista and Escobar, 2021) and in F1 (Ornellas et al., 2016). A study with female rats exposed during gestation to a HF showed an increased preference for positive rewards, such as palatable foods, and elevated anxiety-like behaviors (Camacho et al., 2017).

Food-seeking behavior and anxiety-related behaviors are regulated by the mesolimbic dopaminergic system (Russo and Nestler, 2013; Singh, 2014; Wallace and Fordahl, 2022). Dysregulation of this system can lead to alterations in both feeding behavior and emotional states. Notably, anxiety can influence food-seeking behaviors, as individuals often turn to palatable foods as a coping mechanism to alleviate negative emotional states (Dallman et al., 2003; Adam and Epel, 2007). Parental nutritional status and stress exposure can induce epigenetic modifications in germ cells that alter the expression of genes involved in stress reactivity and reward processing in offspring, thereby programming anxiety and food-seeking phenotypes (Vucetic et al., 2010; Sasaki et al., 2014; Glendining et al., 2018; Freire et al., 2024).

The intergenerational impact of parental high-fat diet on offspring behavior remains poorly understood. In this study, we evaluated F1 progeny derived from F0 parents fed a high-fat diet, assessing food-seeking behavior (toward standard chow and a palatable food) as well as anxiety-like behaviors. We explored sexual dimorphism in the F1 generation and highlighted the influence of parental diet on intergenerational transmission on behavior.

## 2 Materials and methods

### 2.1 General conditions (F0)

Wistar rats aged 21 postnatal days (PN21; 30 females and 30 males; F0) were maintained under a 12:12 light/dark cycle with food and water available *ad libitum*. Rats were randomly assigned to one of two conditions: (1) chow-fed rats (C) and (2) high-fat diet fed rats (HF); each diet was provided *ad libitum* for 18 weeks. Rats were housed in pairs of the same-sex and same diet condition in acrylic cages (23 × 38 × 20 cm) for 18 weeks. Body weight and food consumption were recorded weekly. After 18 weeks of dietary exposure, a series of rats (*C* = 4 males, *C* = 4 females; HF = 8 males and HF = 8 females) were euthanized to collect bilateral Subcutaneous inguinal fat pads (SCAT), Retroperitoneal (RPAT), and Gonadal Adipose Tissue (GAT). Another series of rats (*C* = 8 males, *C* = 8 females; HF = 10 males and HF = 10 females) were bred to produce the F1 offspring cohort (Larqué et al., 2023).

At the end of the feeding paradigm, animals were euthanized via an intraperitoneal overdose of sodium pentobarbital (Pisabental, Aranda; 125 mg/kg; Mexico City, Mexico) administered with a 3 mL syringe and a 21G needle (0.8 × 40 mm). This dose follows the Official Mexican Standard (Nom-033-Sag/Zoo-2014, 2015), which recommends a dose of 2–3 times the intravenous dose (120–150 mg/kg) for euthanasia. Death was confirmed by the absence of heartbeat, respiration, and pedal reflexes for at least 10 min after injection (Leary et al., 1993). The experiments were approved by the Ethics Committee of the Faculty of Medicine at UNAM (FM/DI/013/2018; FM/DI/062/2024).

### 2.2 Diets

Control groups were fed a standard diet (Purina 5001 chow; LabDiet, St. Louis, MO, USA; catalog #5001) containing 59% carbohydrates, 28% protein and 13.4% lipids (energy density: 3.36 kcal/gr). The experimental high-fat diet (HF) was formulated to provide 18% carbohydrates, 20% proteins and 62% of lipids (energy density: 5.41 kcal/gr). In 100 gr, we used lard (17 g) and olive oil (19 ml), supplemented with albumin (15 g), and mixed with standard chow Purina 5001 (49 g) (See Ubaldo-Reyes et al., 2024 for more details of HFD composition).

### 2.3 Mating (F0)

**FIGURE 1:**
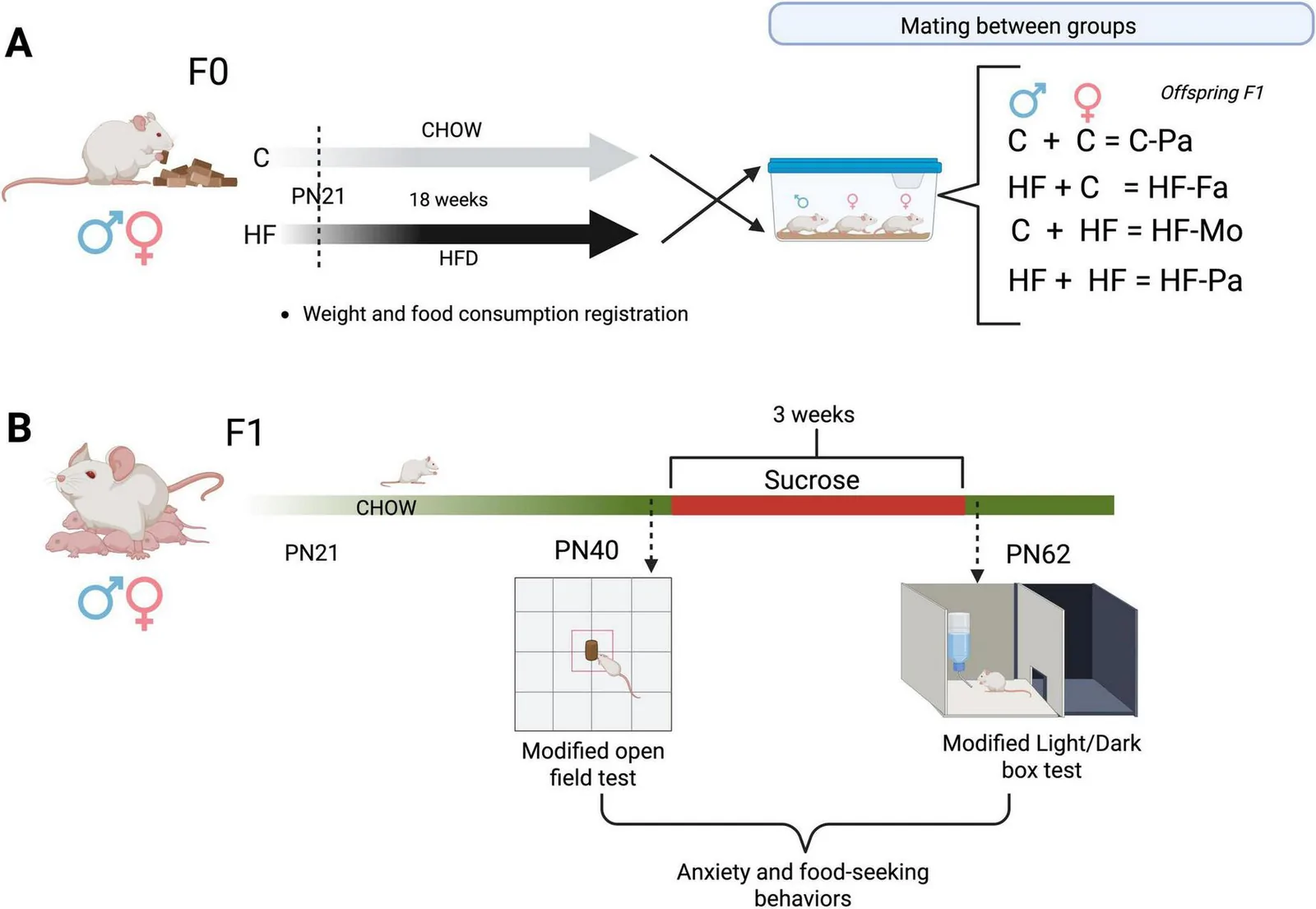
Experimental design. **(A)** F0 female and male rats were exposed to chow diet (C) or high fat diet (HF) for 18 weeks; animals were mated between groups, creating 4 groups in order to obtain F1 generation. **(B)** F1 female and male rats were maintained on a chow diet after weaning and they were evaluated in the open field test on postnatal day 40 (PN40). Then they received a 10% sucrose daily (1 h) for 3 weeks and were exposed to the light/dark box test on postnatal day 62 (PN62). In both tests, parameters for anxiety and food seeking behaviors we evaluated. Created with BioRender.

After 18 weeks of *ad libitum* exposure to the assigned diet, C and HF rats were housed for 1 week, with one male and two females per cage in the next combinations (see Figure 1A): (1) C male and C females; (2) HF male and C females; (3) C male and HF females and (4) HF male and HF female. Reproductive success, pups’ weight, and pup sex were registered. Reproductive success was defined as (1) the proportion of pregnant females after 1 week of mating opportunity and (2) the number of newborn pups found in the nest on postnatal day 1 (PN1).

### 2.4 Offspring (F1)

Offspring were obtained from the combination of previous F0 mating, generating four groups named: (1) Pups from C father and C mother (C-Pa); (2) Pups from HF father and C mother (HF-Fa); (3) Pups from C father and HF mother (HF-Mo) and (4) Pups from HF father and HF mother (HF-Pa). At birth [postnatal day 0 (PN0)], litters born from the same dietary condition were culled to standardize litter size to eight pups. During lactation, the mothers were fed the corresponding diet, but at weaning all F1 offspring were fed exclusively a standard chow diet until the end of the study (see Figure 1B). At PN21 three males (M) and three females (F) from each litter were selected randomly to form the experimental groups of the F1 generation, as follows: C-Pa (*n* = 8), HF-Mo (*n* = 12), HF-Fa (*n* = 12), and HF-Pa (*n* = 12). From weaning onward, rats were housed in groups (*n* = 4) from the same dietary background and of the same sex. Body weight and food consumption were monitored and recorded weekly throughout the entire experiment.

### 2.5 Modified open field test (MOFT)

At 40 days of age, the offspring were evaluated in a modified open field test adapted to evaluate the animals’ motivation to seek-food in a novel and anxiogenic environment. The open field was a white acrylic arena (60 × 60 × 30 cm) illuminated with bright light (390 lux). To assess food-seeking motivation, a single standard food pellet was securely fixed to the center of the arena using a small drop of hot silicone. All animals were food-deprived for 12 h prior to testing to ensure motivation for food. The day of the test, they were placed individually in the MOFT and recorded for 5 min. Behavior during the 5 min test was recorded with a camera on top of the arena. The arena floor was divided into 16 equal squares (4 × 4 grid) for scoring ambulation and entries to the center (the central 4 squares). Specific criteria were used to evaluate food-seeking behavior and anxiety-like behaviors (see Supplementary Table 1). The corresponding criteria were double-blind evaluated by three independent researchers who were blind to the experimental conditions, and inter-rater reliability was > 90% (Gutiérrez-Pérez et al., 2023; González-González et al., 2024; Cuellar-Salazar et al., 2026).

### 2.6 Palatable food exposition

One day after the MOFT (PN41), all F1 groups were exposed to a 10% sucrose solution for 1 h daily at 10:00 h (4 h after lights on) for 3 weeks. Sucrose consumption was monitored weekly measuring the milliliters consumed by each rat. Access to regular chow and tap water were maintained *ad libitum*.

### 2.7 Light/dark box test (L/D-BT)

After 3 weeks of daily scheduled sucrose exposure, rats were individually tested in the light/dark box test to assess their motivation to consume the sucrose solution in a novel and anxiogenic environment. The apparatus (50 × 36 × 33 cm) was divided into two compartments of equal size (25 × 36 × 33 cm each): a brightly illuminated white compartment (563 lux) and a dark, enclosed compartment (1 lux) (Desikan et al., 2008). The two compartments were connected by a central guillotine door (10 × 10 cm). At the far end of the illuminated compartment, directly opposite the central door, a bottle containing the 10% sucrose solution was placed to ensure its visibility to the animal. At the start of the trial, the rat was placed in the light compartment with the door closed for a 30-s habituation interval, allowing it to acclimate and visualize the sucrose bottle. After this interval, the door was raised, permitting the animal to move freely between the compartments for a 5-min test session. The session was recorder with a camera placed on top of the light/dark box. To prevent olfactory cues, the entire apparatus was thoroughly cleaned with 70% ethanol and dried between each test session. Specific criteria were used to evaluate food-seeking behavior and anxiety-like behaviors (see Supplementary Table 1). These behavioral criteria are validated in the literature (Blasco-serra et al., 2017) and were double-blind evaluated by three independent researchers who were blind to the experimental conditions, and inter-rater reliability was > 90%.

### 2.8 Statistics

Statistical analyses were performed using GraphPad Prism version 10.0 (GraphPad Software, San Diego, CA, USA). Statistical power for the sample size, we used http://www2.ccrb.cuhk.edu.hk/stat/mean/osm_equality.htm with the following parameters: power (1-β) = 0.80, α = 0.05, and an allowable difference = 0.4, and populations variance = 1. The power analysis indicated that *n* = 8 per group would provide adequate power (≥ 80%) to detect significant differences. The statistics were used when data reached the assumptions of normality and homoscedasticity. When these assumptions were not reached, non-parametric statistics were used. Two-way ANOVA for repeated measures was used for longitudinal measures including body weight gain, food intake (kilocalories and grams) and sucrose intake (in both F0 and F1) over the 3 weeks. A *t*-test was used to compare the number of pups per litter between conditions, and adipose tissue measurements in the F0 generation. Two-way ANOVA was used to analyze birth and weaning weight in the F1 generation. One-way ANOVA was applied to assess body weight and food consumption in male and female F1 offspring at postnatal day 40 (PN40), total sucrose intake, and all parameters for food-seeking and anxiety-like behavior in each test. Exceptions were entries to the center zone, entries to the pellet square, ambulation, and fecal boli in the MOFT, as well as transitions in the L/D-BT, which were analyzed using the Kruskal-Wallis test. For all behavior evaluated for food-seeking behavior and anxiety-like behavior, statistically significant differences from the control group were identified. The direction of these differences (i.e., an increase or decrease in a given measure) was interpreted as indicative of heightened or diminished motivation to seek the respective food reward or anxiety-like behavior, as demonstrated in Supplementary Table 1.

## 3 Results

### 3.1 F0 Body weight, food intake and withe adipose tissue

**FIGURE 2:**
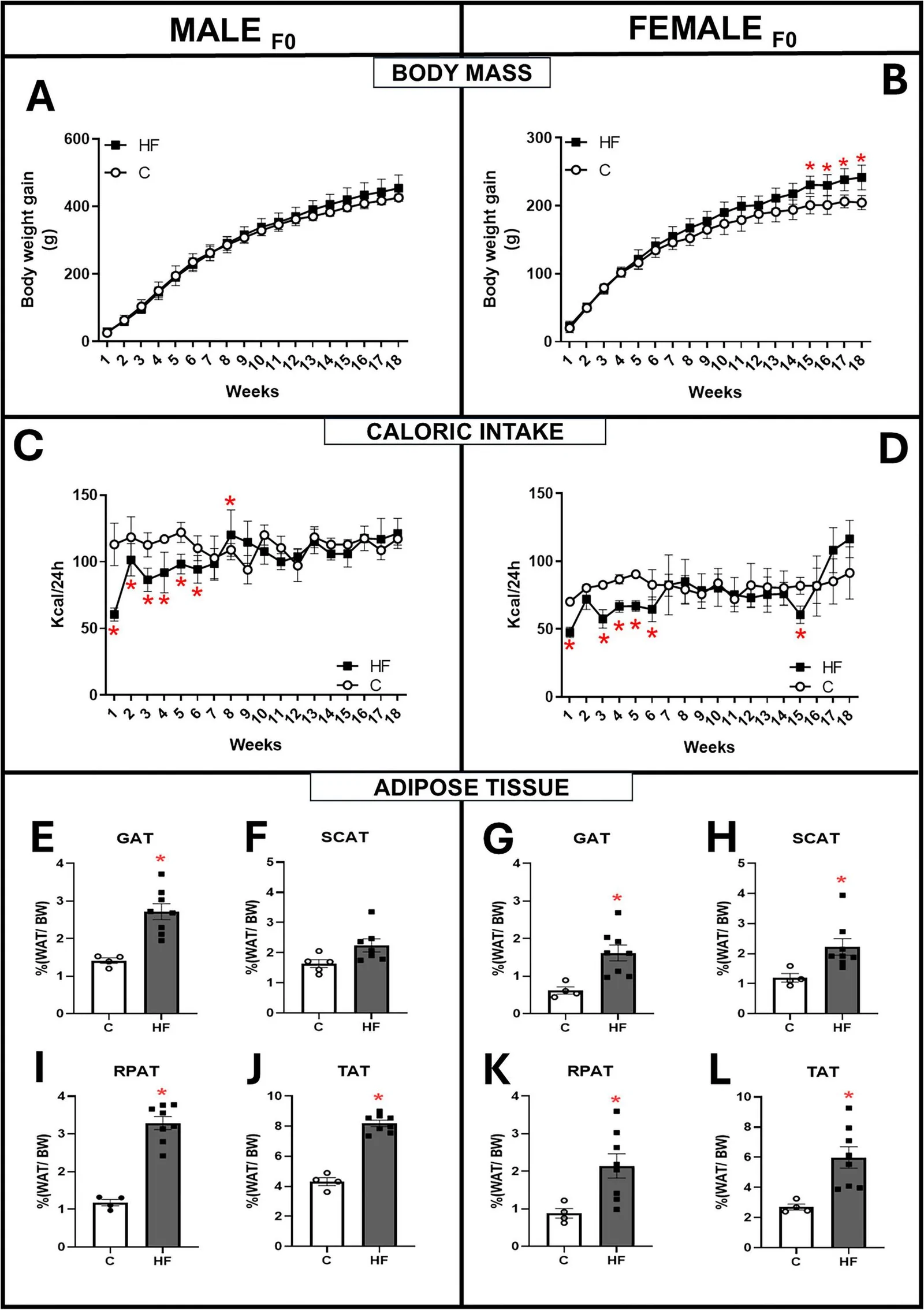
Body mass, caloric intake and adipose tissue from F0 male and female rats fed with chow diet (°) or high-fat diet (◼). **(A)** Male and **(B)** female body weight gain for 18 week. **(C)** Male and **(D)** female 24 h kilocalorie consumption for 18 weeks. Male and female adipose tissue expressed as percentage of total body weight: **(E)** Male and **(G)** female Gonadal Adipose Tissue (GAT); **(F)** Male and **(H)** female Subcutaneous Adipose Tissue (SCAT); **(I)** Male and **(K)** female Retroperitoneal Adipose Tissue (RPAT); **(J)** Male and **(L)** female Total Adipose Tissue (TAT). Data are shown as the mean ± SEM. Asterisk (*) indicates statistical differences from the CD group (*p* < 0.05).

All rats have increased body weight over the 18 weeks of diet exposure. In male rats (Figure 2A), the two-way ANOVA for repeated measures (RM ANOVA) indicated significant effects for the interaction of diets X time [*F*_(17_,_272)_ = 3.61; *p* < 0.0001]. Likewise, in female rats (Figure 2B), the RM ANOVA indicated significant effects for the interaction of diets X time [*F*_(17_,_272)_ = 10.2; *p* < 0.0001].

For diet consumption over 18 weeks, the RM ANOVA indicated for males (Figure 2C) a significant effect for the interaction of diets X time [*F*_(17_,_272)_ = 14.63; *p* < 0.0001], as well as for females (Figure 2D), [*F*_(17_,_272)_ = 9.39; *p* < 0.0001]. Importantly, this difference was based on a reduction in caloric intake in both HF groups in weeks 1 to 6.

Despite not observing significant differences body weight gain, after 18 weeks of diet, HF-males exhibited higher accumulation of GAT (*t* = 4.21, df = 10, *p* = 0.0018; Figure 2E), RPAT (*t* = 8.17, df = 10, *p* < 0.0001; Figure 2I), and TAT (*t* = 11.11, df = 10, *p* < 0.0001; Figure 2J) obtained as a proportion of the total body weight; no differences were found in SCAT (*t* = 1.82, df = 10, *p* = NP; Figure 2F). HF-females exhibited a higher accumulation in GAT (*t* = 3.22, df = 10, *p* = 0.009; Figure 2G), SCAT (*t* = 2.5, df = 10, *p* = 0.031; Figure 2H), RPAT (*t* = 2.65, df = 10, *p* = 0.024; Figure 2K) and in TAT (*t* = 3.17, df = 10, *p* = 0.009; Figure 2L) obtained as a proportion of the total body weight.

Notably, examination of the magnitude of adipose tissue expansion revealed that HFD-fed females showed approximately a 2-fold increase in subcutaneous adiposity and a 3-fold increase in gonadal adiposity relative to their chow-fed controls, whereas the magnitude of adipose tissue expansion was comparatively smaller in males. This disproportionate adipose tissue expansion in females likely contributed to the significant body weight gain observed exclusively in HFD-fed females.

### 3.2 Reproductive success and pups

75% of C males were reproductive compared to 80% of HF males (see Supplementary Figure 1A); while 75% of C females were successful compared to 90% of HF females (see Supplementary Figure 1B). The number of pups found per litter on day PN1 was not different between dietary conditions of each litter, and the *t*-test did not show significant differences in pups from males (*t* = 0.08, df = 12, *p* = NP; Supplementary Figure 1C), neither in pups from females (*t* = 1.564, df = 13, *p* = NP; Supplementary Figure 1D). The average litter weight was calculated at birth and weaning. A two-way ANOVA (diet X offspring sex) showed no main effect of maternal or paternal diet on litter weight either at birth (Supplementary Figures 1E, F) or weaning (Supplementary Figures 1G, H).

### 3.3 Body weight and food intake of F1 offspring

**FIGURE 3:**
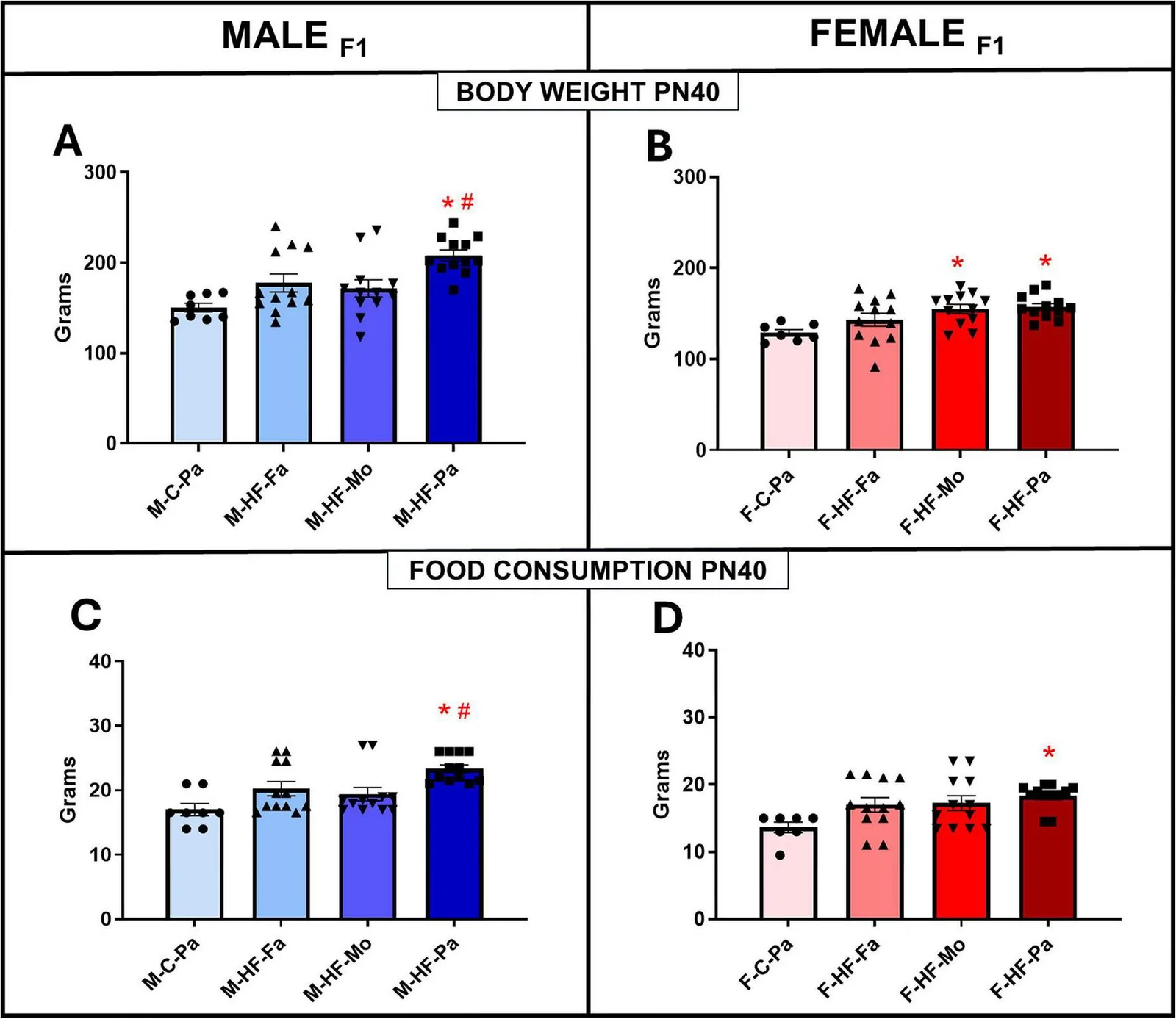
Body weight at postnatal 40 (P40) of male **(A)** and female **(B)** offspring born from control parents (C-Pa; ∙), HF father (HF-Fa;▲), HF mother (HF-Mo;▼) or both HF parents (HF-Pa; ◼). Chow diet consumption at postnatal 40 (P40) of males **(C)** and female **(D)** offspring from C-Pa, HF-Fa, HF-Mo or HF-Pa. Data are shown as the mean ± SEM. Asterisks (*) indicate a significant difference from Ctrl-Pa and (#) indicate a significant difference from HF-Mo (*p* < 0.05).

At PN40, body weight and the 24 h food consumption were evaluated in the F1 offspring. In males the one-way ANOVA indicated statistically significant differences among the groups [*F*_(3_,_40)_ = 7.41; *p* = 0.0005; Figure 3A], with a higher body weight for M-HF-Pa as compared with M-C-Pa and M-HF-Mo. Likewise, in female subjects, the one-way ANOVA showed statistically significant differences [*F*_(3_,_39)_ = 4.56; *p* = 0.0078; Figure 3B] among groups, with the highest body weight for the F-HF-Pa group.

Both M-HF-Pa and F-HF-Pa displayed higher food consumption compared to offspring of control parents (C-Pa). In males, the one-way ANOVA showed statistically significant differences among male groups [*F*_(3_,_40)_ = 6.78; *p* = 0.0008; Figure 3C]; and females [*F*_(3_,_39)_ = 3.44; *p* = 0.02; Figure 3D].

### 3.4 Food-seeking behavior for chow food in F1 (MOFT)

The MOFT was used to evaluate behavior associated with food-seeking and anxiety-like behavior in an anxiogenic environment. It is important to note that not all rats exhibited food-seeking behaviors evaluated. Therefore, as a preliminary analysis we obtained a table with the percentage of rats per group that presented each evaluated behavior and compared with the C-Pa group to determine whether there were more or fewer subjects in order to know if the subjects increased or decreased by each condition per group. For food-seeking behaviors, males and females that interacted with the pellet in the center of the MOFT increased in percentage in all groups with one or both parents were fed with HF (See Supplementary Table 2).

When comparing the values obtained by males group, regardless of whether of subjects that presented the behavior increased or decreased, significant differences in the latency of interaction with the pellet [*F*_(3_,_31)_ = 6.31; *p* = 0.001; Table 1] were observed, where M-HF-Pa showed increased latency compared to M-C-Pa and M-HF-Mo. Additionally, significant differences in the total time of interaction with the pellet [*F*_(3_,_31)_ = 3.21; *p* = 0.03; Table 1] were found, where M-HF-Pa showed decreased time compared to M-C-Pa. No differences were observed in the entries to the pellet square.

**TABLE 1:** Male motivational criteria to evaluate food-seeking behavior for chow and anxiety-like behaviors.

Criteria in the MOFT	M-C-Pa	M-HF-Fa	M-HF-Mo	M-HF-Pa
	$\bar{X}$ or x̃ (SEM/min-max)	$\bar{X}$ or x̃ (SEM/min-max)	$\bar{X}$ or x̃ (SEM/min-max)	$\bar{X}$ or x̃ (SEM/min-max)
Food seeking behavior				
Entries to the pellet zone (*n*)	1 (0–7)	3.5 (0–7)	4.5 (0–7)	4 (0–7)
Latency to interact with the pellet (s)	3.05 (± 0.22)	46.03 (± 14.79)	18.85 (± 8.97)	83.11 (± 12.82)*#
Interaction with the pellet (s)	30.41 (± 16.0)	10.95 (± 2.78)	18.01 (± 4.84)	5.31 (± 0.68)*
Anxiety-like behavior				
Ambulation (*n*)	87.5 (55–125)	103.5 (46–142)	104 (78–167)	117 (75–201)
Entries to the center (*n*)	9.5 (0–37)	24.5 (4–36)	28 (4–49)	26 (3–44)
Grooming (s)	44.25 (± 9.67)	37.42 (± 6.7)	33.89 (± 8.13)	15.41 (± 4.35)
Fecal boli (*n*)	0 (0–2)	0.5 (0–4)	1 (0–4)	0 (0–6)

Male offspring (M) from control parent (C-Pa), HF father (HF-Fa), HF mother (HF-Mo) or HF parents (HF-Pa) exposed to the Modified Open Field Test (MOFT). Criteria: n = number of behaviors; s = seconds. Data are shown as the mean ± SEM or the median ± min-max. Asterisk (*) indicates a significant difference from C-Pa and (#) indicates a significant difference from HF-Mo (*p* < 0.05). Numbers in bold indicate significant differences.

When comparing the values obtained in female groups, regardless of whether the number of subjects that presented the behavior increased or decreased, we observed significant differences in the latency of interaction with the pellet [*F*_(3_,_36)_ = 5.2; *p* = 0.004; see Table 2], where F-HF-Pa showed increased latency compared to all groups. Furthermore, significant differences in the entries to the pellet square (H = 10.09; *p* = 0.01) were found, where F-HF-Fa showed more entries that the F-C-Pa group (see Table 2). No significant differences were found in the total time of interaction with the pellet.

**TABLE 2:** Female motivational criteria to evaluate food-seeking behavior for chow and anxiety-like behaviors.

Criteria in the MOFT	F-C-Pa	F-HF-Fa	F-HF-Mo	F-HF-Pa
	$\bar{X}$ or x̃ (SEM/min-max)	$\bar{X}$ or x̃ (SEM/min-max)	$\bar{X}$ or x̃ (SEM/min-max)	$\bar{X}$ or x̃ (SEM/min-max)
Motivational parameters				
Entries to the pellet square (*n*)	1.5 (0–5)	5.5 (0–15)*	5 (1–12)	5 (1–8)
Latency to interact with the pellet (s)	47.98 (± 12.11)	51.4 (± 12.95)	47.83 (± 11.0)	113.79 (± 18.39)*#%
Interaction with the pellet (s)	28.69 (± 20.72)	41.65 (± 14.74)	22.94 (± 7.49)	4.79 (± 0.49)
Anxiety parameters				
Ambulation (*n*)	83.5 (46–107)	112.5 (65–210)	140 (49–192)*	153 (120–220)*
Entries to the center (*n*)	7 (6–26)	33.5 (9–63)*	32 (3–46)	34 (7–59)*
Grooming (s)	38.1 (± 4.76)	25.36 (± 6.24)	21.71 (± 5.47)	8.44 (± 1.41)*
Fecal boli (*n*)	0 (0–3)	0 (0–3)	0 (0–4)	0 (0–4)

Female offspring (F) from control parent (C-Pa), HF father (HF-Fa), HF mother (HF-Mo) or HF parents (HF-Pa) exposed to the Modified Open Field Test (MOFT). Criteria: n = number of behaviors; s = seconds. Data are shown as the mean ± SEM or the median ± min-max. Asterisks (*) indicates a significant difference from C-Pa; (%) indicates a significant difference from HF-Fa (#) indicates a significant difference from HF-Mo (*p* < 0.05). Numbers in bold indicate significant differences.

### 3.5 Anxiety-like behavior in F1 (MOFT)

When analyzing parameters used to evaluate anxiety in the MOFT in males (F1), we only observed a decrease percentage of subjects in the of M-HF-Pa group (91.6%) compared to M-C-Pa (100%; see Supplementary Table 2) in grooming. No statistical differences among groups were found in ambulation, entries to the center, grooming or fecal boli (see Table 1), which reflect no anxiety-related changes.

In females F1 offspring, a decrease in the percentage of subjects in the F-HF-Pa group compared to M-C-Pa (See Supplementary Table 2) was observed in grooming. However, unlike males, the F-HF-Mo and F-HF-Pa groups increased the number of visited squares (ambulation) compared to the F-C-Pa group (H = 14.04; *p* = 0.002). Additionally, F-HF-Fa and F-HF-Pa increased entries to the center (9 squares) compared to the F-C-Pa group (H = 12.28; *p* = 0.006). Finally, F-HF-Pa decrease their time in grooming compared to the F-C-Pa group [*F*_(3_,_39)_ = 5.18; *p* = 0.004]. All this data shows a reduced anxiety across several parameters in the F-HF-Pa group.

### 3.6 Motivation for sucrose consumption

**FIGURE 4:**
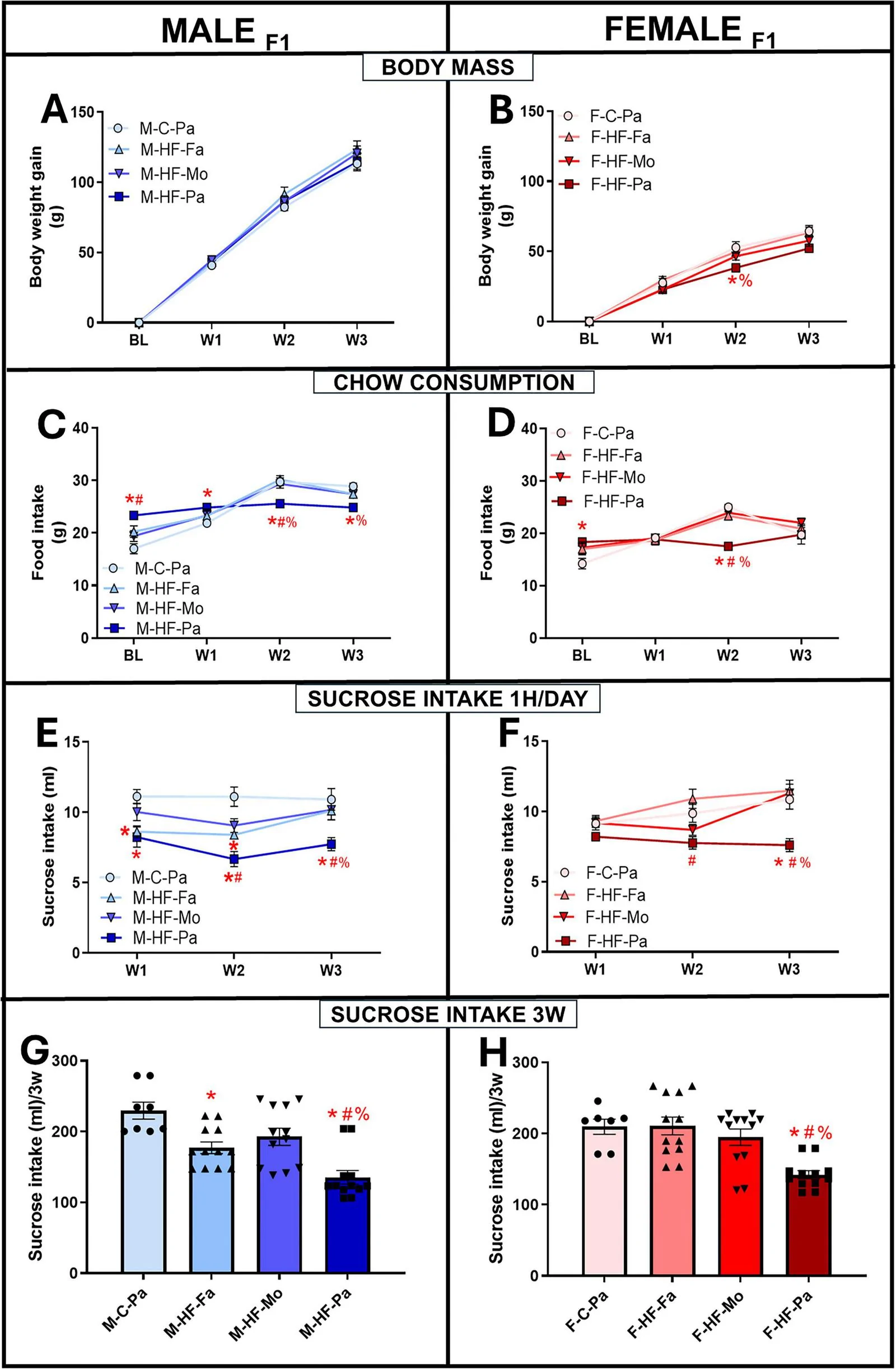
Sucrose administration in F1. Body weight gain of male [M; **(A)**] or female [F; **(B)**]offspring from control parent (C-Pa; ∙), HF father (HF-Fa;▲), HF mother (HF-Mo;▼) or both HF parents (HF-Pa; ◼). during daily sucrose administration. Chow diet consumption of M **(C)** or F **(D)** offspring from C-Pa, HF-Fa, HF-Mo or HF-Pa during daily sucrose administration. 10% sucrose consumption of M **(E)** or F **(F)** offspring from C-Pa, HF-Fa, HF-Mo or HF-Pa along 3 weeks. Total sucrose intake along 3 weeks of M **(G)** or F **(H)** offspring from C-Pa, HF-Fa, HF-Mo or HF-Pa. BL (baseline), W1 (week 1), W2 (week 2), W3 (week 3). Data are shown as the mean ± SEM Asterisks (*) indicates a significant difference from C-Pa; (%) indicates a significant difference from HF-Fa (#) indicates a significant difference from HF-Mo (*p* < 0.05).

During the three-weeks sucrose access period, body weight gain was monitored. In males, the RM ANOVA showed significant differences in the factor time [*F*_(2_,_84)_ = 1128; *p* < 0.0001; Figure 4A], but not in the factor “parental diet” nor in the interaction. For females, the RM ANOVA showed significant differences in interaction (parental diet X time) [*F*_(9_,_117)_ = 2.51; *p* = 0.011; Figure 4B] and the *post hoc* test indicated that F-HF-Pa gained less weight in week 2 when compared to F-C-Pa and F-HF-Fa groups.

At the same time, chow consumption was registered once a week. In males, the RM ANOVA showed significant differences in interaction (parental diet X time) [*F*_(9_,_120)_ = 10.01; *p* < 0.0001; Figure 4C], showing that at baseline (BL) and in week 1, M-HF-Pa overconsumed chow pellets, and in week 2 and 3 consumed less than the other groups. In females subjects, the RM ANOVA showed significant differences in interaction (parental diet X time) [*F*_(9_,_117)_ = 6.66; *p* < 0.0001; Figure 4D], and we observed that in week 2, F-HF-Pa consumed fewer chow pellets than the other groups.

The 10% sucrose solution was administrated 1 h daily and was evaluated. In males, the RM ANOVA did not show significant differences in interaction (parental diet X time), but it did in time factor [*F*_(2_,_68)_ = 6.07; *p* = 0.005] and in parental diet factor [*F*_(3_,_40)_ = 8.23; *p* = 0.0002]. Surprisingly, all groups with one or both parents fed with HF consumed less sucrose than control offspring. Specifically, M-HF-Pa consumed less sucrose over the 3 weeks, while M-HF-Fa consumed less only in week 1 and 2 compared to M-C-Pa (see Figure 4E). The total consumption of sucrose water over the 3 weeks was summed per group and it was observe that M-HF-Pa was the group that consumed less than all other groups, while M-HF-Fa only consumed less than M-C-Pa. The one-way ANOVA showed statistically significant differences [*F*_(3_,_40)_ = 12.52; *p* < 0.0001; Figure 4G].

In females, the RM ANOVA showed significant differences in the interaction (parental diet X time) [*F*_(6_,_78)_ = 5.62; *p* < 0.0001]. As in males, F-HF-Pa consumed less sucrose than F-C-Pa in week 3. Additionally, in week 2, F-HF-Fa overconsumed sucrose compared to F-HF-Pa (see Figure 4F). Like in males, female offspring from both parents fed with HF consumed less sucrose water than all other groups. The one-way ANOVA showed statistically significant differences [*F*_(3_,_39)_ = 9.65; *p* < 0.0001; Figure 4H].

### 3.7 Food-seeking behavior for palatable food in F1 (L/D-BT)

Not all animals exhibited all the behaviors evaluated in the L/D-BT. In males, HF-Fa and HF-Pa groups showed a reduced percentage of subjects that presented the evaluated behaviors compared to the control group (100%; see Supplementary Table 3). The one-way ANOVA showed statistical differences in the latency to drink sucrose [*F*_(3_,_35)_ = 5.79; *p* = 0.002], with increased time to drink in M-HF-Pa compared to M-C-Pa and M-HF-Mo. Furthermore, M-HF-Pa showed a reduction in time interacting with the sucrose bottle [*F*_(3_,_38)_ = 3.27; *p* = 0.03], but no statistical differences were found in sucrose intake, nor in percentage of preference for the light compartment (see Table 3).

**TABLE 3:** Male motivational criteria to evaluate food-seeking behavior for sucrose and anxiety-like behaviors.

Criteria in the L/D-BT	M-C-Pa	M-HF-Fa	M-HF-Mo	M-HF-Pa
	$\bar{X}$ or x̃ (SEM/min-max)	$\bar{X}$ or x̃ (SEM/min-max)	$\bar{X}$ or x̃ (SEM/min-max)	$\bar{X}$ or x̃ (SEM/min-max)
Motivational parameters				
Preference for light compartment (%)	55.71 (± 6.88)	55.19 (± 7.84)	66.4 (± 4.81)	48.6 (± 4.72)
Latency to drink sucrose bottle (s)	58.75 (± 15.21)	72.87 (± 25.1)	36.87 (± 10.2)	150.77 (± 28.82)*#
Interaction with sucrose bottle (s)	126.81 (± 24.24)	113.54 (± 22.87)	134.23 (± 20.23)	**56.04 (± 11.47)#**
Sucrose intake (ml)	4.12 (± 0.87)	3.95 (± 0.88)	4 (± 0.55)	1.95 (± 0.51)
Anxiety parameters				
Transitions between compartments (*n*)	9 (4–15)	10 (1–18)	11 (7–17)	8 (1–6)
Latency to enter the dark compartment (s)	15.37 (± 8.21)	11.34 (± 3.23)	15.05 (± 5.5)	**39.99 (± 10.39)#**

Male offspring (M) from control parent (C-Pa), HF father (HF-Fa), HF mother (HF-Mo) or HF parents (HF-Pa) exposed to Ligth/Dark Box Test (L/D-BT). Criteria: n = number of behaviors; s = seconds;% = percentage; ml = milliliters. Data are shown as the mean ± SEM. or the median ± min-max. Asterisks (*) indicates a significant difference from C-Pa and (#) indicates a significant difference from HF-Mo (*p* < 0.05). Numbers in bold indicate significant differences.

In female offspring, regarding the percentage of subjects per group that presented the evaluated behaviors, HF-Fa and HF-Mo groups increased compared to C-Pa group, while HF-Pa decreased (see Supplementary Table 3). The one-way ANOVA showed statistical differences in the preference index for being in the light compartment [*F*_(3_,_38)_ = 3.66; *p* = 0.02], demonstrating that F-HF-Pa had a reduction in preference compared to F-C-Pa. Moreover, for F-HF-Pa, statistical differences were observed in the latency to drink sucrose [*F*_(3_,_34)_ = 6.56; *p* = 0.001], a reduction in time interacting with the sucrose bottle [*F*_(3_,_36)_ = 11.27; *p* < 0.0001], and lower sucrose intake [*F*_(3_,_38)_ = 8.32; *p* = 0.0002; see Table 4] compared to all other groups. F-HF-Mo also showed a reduction in time interacting with the sucrose bottle compared to F-Ctrl-Pa.

**TABLE 4:** Female motivational criteria to evaluate food-seeking behavior for sucrose and anxiety-like behaviors.

Criteria in the L/D-BT	F-C-Pa	F-HF-Fa	F-HF-Mo	F-HF-Pa
	$\bar{X}$ or x̃ (SEM/min-max)	$\bar{X}$ or x̃ (SEM/min-max)	$\bar{X}$ or x̃ (SEM/min-max)	$\bar{X}$ or x̃ (SEM/min-max)
Motivational parameters				
Preference for light compartment (%)	59.6 (± 5.61)	53.13 (± 7.31)	44.3 (± 5.0)	30.9 (± 5.78)*
Latency to drink sucrose bottle (s)	49.07 (± 18.24)	79.18 (± 23.9)	105.14 (± 28.18)	210.29 (± 19.21)*#%
Interaction with sucrose bottle (s)	129.78 (± 12.0)	112.07 (± 18.98)	71.72 (± 13.3)*	18.78 (± 4.98)*#%
Sucrose intake (ml)	3.57 (± 0.52)	3.87 (± 0.7)	2.58 (± 0.41)	0.63 (± 0.18)*#%
Anxiety parameters				
Transitions between compartments (*n*)	15 (8–18)	9 (2–24)	11.5 (2–21)	6 (1–13)*
Latency to enter the dark compartment (s)	8.42 (± 2.47)	15.45 (± 6.65)	15.86 (± 5.67)	34.14 (± 10.05)

Female offspring (F) from control parent (C-Pa), HF father (HF-Fa), HF mother (HF-Mo) or HF parents (HF-Pa) exposed to Ligth/Dark Box Test (L/D-BT). Criteria: n = number of behaviors; s = seconds;% = percentage; ml = milliliters. Data are shown as the mean ± SEM or the median ± min-max. Asterisks (*) indicates a significant difference from C-Pa; (%) indicates a significant difference from HF-Fa (#) indicates a significant difference from HF-Mo (*p* < 0.05). Numbers in bold indicate significant differences.

### 3.8 Anxiety-like behavior in F1 (L/D-BT)

When analyzing parameters to evaluate anxiety in males F1 offspring, an increase on latency in M-HF-Pa to enter the dark box in compared to M-HF-Mo was observed [*F*_(3_,_40)_ = 3.48; *p* = 0.02; see Table 3], whit no changes in the number of transitions between compartments. When analyzing females F1 offspring, we observed a decrease in the number of transitions between light and dark compartments in F-HF-Pa compared to F-Ctrl-Pa (H = 9.84; *p* = 0.02), with no changes in the latency to enter the dark compartment (see Table 4).

## 4 Discussion

This study demonstrates that parental consumption of HF significantly influences behavioral phenotypes in offspring, with the most pronounced effects observed when both parents were exposed to HF. Our findings reveal that offspring of HF-fed parents exhibit increased body weight, hyperphagia, reduced anxiety-like behaviors, and diminished motivation for palatable foods, mostly in females. Also, maternal exposure to HF exhibited moderate and sex-dependent effects. Overall, the maternal-only affect female F1 body weight and some behavioral results. These results underscore the role of parental diet as an intergenerational risk factor capable of reprogramming neurobehavioral circuits related to energy balance and reward processing.

### 4.1 Sex-dependent effects on body weight and fat accumulation in parents (F0)

After 18 weeks exposed to a HF, only F0 females exhibited a significant increase in body weight gain compared to their chow controls. However, in both males and females, a significant increase in adipose tissue was observed, indicating a dysregulation in lipid metabolism. A previous study with chronic exposure to HF in two models of rats: obesity-resistant S5B/Pl (S5B) and obesity-prone Osborne-Mendel (OM) rats found that males (S5B and OM) and OM females increased body weight; and that visceral and subcutaneous adipose tissue was higher in male rats, also males reached criteria of metabolic syndrome while females did not (Poret et al., 2021). Our data agree with a study that demonstrated that male and female Wistar rats exposed to chronic HF consumption did not develop a higher body weight, but developed increased adiposity, especially in males (Estrany et al., 2011). These findings suggest that increased adiposity and metabolic dysregulation can occur without proportional increases in body weight and with sexual dimorphism. Moreover, they highlight the importance of assessing adipose tissue depots and metabolic markers beyond simple weight measurements (Macotela et al., 2009; Estrany et al., 2011).

We should highlight that our data revealed that HFD-fed females showed approximately a 2-fold increase in subcutaneous adiposity and a 3-fold increase in gonadal adiposity relative to their chow-fed controls, whereas the magnitude of adipose tissue expansion was comparatively smaller in males. Consequently, only females reached a statistically significant increase in body weight gain relative to their controls. A possible explanation for this sexual dimorphism lies in the well-described sex differences in adipose tissue biology. Both human females and female rodents generally exhibit greater adipose tissue expandability, particularly in subcutaneous depots, a process largely regulated by estrogens (Palmer and Clegg, 2015). Estrogens have been postulated to promote adipocyte hyperplasia and lipid storage while maintaining the metabolic flexibility of adipose tissue, defined as the capacity to appropriately switch between lipid storage and mobilization in response to fed and fasting states (Karastergiou et al., 2012). In contrast, males tend to present greater visceral fat accumulation and develop adipose tissue dysfunction earlier under metabolic stress such as HFD exposure (Palmer and Clegg, 2015). Thus, differences in adipose tissue distribution and expansion capacity may contribute to the greater body weight gain observed in HFD-fed females.

### 4.2 Lack of detrimental effects on fertility in F0 rats fed a chronic high-fat diet

Regarding F0, previous studies suggest that paternal obesity induced by chronic HF consumption can lead to subfertility, with detrimental effects on gamete quality, hormonal regulation, and reproductive outcomes (Binder et al., 2012; Fullston et al., 2012). Similarly, maternal obesity induced by HF consumption may compromise female reproductive function through multiple mechanisms, including ovarian dysfunction, disrupted estrous cyclicity, and altered uterine receptivity (Jungheim et al., 2010; Bazzano et al., 2015). In our study, group size differences were standardized using percentage-bases rates, with each group’s n as 100%. We assessed two operational definitions of reproductive success: (1) the proportion of pregnant females after 1 week of mating opportunity, and (2) the number of newborn pups found in the nest on postnatal day 1 (PN1). We found that males and females consuming a HF diet had comparable or slightly higher reproductive success (C males 75% vs. HF males 80%; C females 75% vs. HF females 90%) as measured by pregnancy rates, and the number of pups per litter did not differ between dietary groups. However, we acknowledge important limitations in interpreting these findings. First, while litter size is a commonly used proxy for reproductive success, it does not capture other critical parameters such as pup viability, gestational length, or long-term offspring health. A larger litter does not necessarily indicate better reproductive fitness, as it may come at the cost of reduced maternal resources per pup (Crean and Senior, 2019). Second, our study did not assess estrous cyclicity, hormonal profiles (e.g., gonadotropins, sex steroids), gamete quality, or molecular markers of reproductive function that would provide mechanistic insight into reproductive outcomes. However, the reproductive consequences of HF may depend on several factors, including the duration of dietary exposure, the specific composition of the diet, and the magnitude of weight gain achieved. Thus, the variability in reproductive outcomes across studies underscores the importance of considering dietary protocols, metabolic status, and species- or strain-specific factors when evaluating the effects of HF on fertility (Crean and Senior, 2019; Sureshchandra et al., 2019). Future studies incorporating comprehensive reproductive assessments, including hormonal measurements, gamete quality analysis, and detailed fertility indices, are needed to definitively determine whether chronic HF consumption impairs reproductive function in this model.

### 4.3 High-Fat diet parental effects in offspring: body weight and food consumption (F1)

Consistent with previous reports, in the F1, the combination of maternal with paternal HF exposure led to increased body weight in adolescent male and female offspring (P40). Maternal HF influenced body weight in female offspring. Present results are consistent with the study by Ornellas et al. (2016), which demonstrates that the combined effect of parental obesity (more than single parent-obesity) promotes hyperphagia and increased adiposity in mouse offspring (Ornellas et al., 2016). The intergenerational effects of parental HF on offspring metabolic health have been extensively documented, with both maternal and paternal nutrition contributing independently and additively to obesity risk (Dunn and Bale, 2011; Fullston et al., 2013). In particular, maternal HF exposure is associated with alterations in hypothalamic appetite regulation and adipocyte development in offspring, with effects that are often more pronounced in females (Dearden and Ozanne, 2015). Similarly, paternal HF consumption has been linked to epigenetic modifications in sperm that transmit metabolic phenotypes to offspring, which may synergize with maternal effects when both parents are exposed (Fullston et al., 2013; Lane et al., 2015). The present study showed that when both parents consumed HF, offspring of both sexes exhibited marked increases in body weight and chow consumption (hyperphagia) during adolescence. This aligns with the DOHaD hypothesis, which posits that parental nutritional status can program offspring physiology through epigenetic and other non-genetic mechanisms (Barker, 2007; Waterland and Michels, 2007). Since our F1 cohort was designed for behavioral assessment, we reference Ornellas et al. (2016), a comparable study providing robust adiposity and hyperphagia data in offspring. However, it is important to note that we did not directly measure adiposity in the F1 offspring, as our primary focus was on behavioral outcomes. Future studies should include comprehensive metabolic phenotyping, including measures of adipose tissue mass, glucose homeostasis, and inflammatory markers, to fully characterize the metabolic consequences of parental HF exposure in offspring.

### 4.4 High-fat parental effects in offspring: reduction in food-seeking and anxiety-like behavior (F1)

In a study where animals were exposed to 10% sucrose solution, HF-Pa offspring (males and females) showed reduced motivation for palatable food (also M-HF -Fa-), as indicated by lower sucrose consumption along 3 weeks. The reduced motivation agrees with present results obtained with both behavioral tests (MOFT and L/D-BT) in which we show that offspring from HF-Pa were the most affected; males obtained consistent 4 of 7 parameter evaluated and females obtaining 5 of 7 parameter evaluated, showing reduced food-seeking behavior for chow food and for palatable food. It is important to note that after performing the MOFT we did not measure food consumption, as recommended in Novelty Suppressed-Feeding Test (Blasco-serra et al., 2017). The reduced motivation for sucrose observed in HF-Pa offspring, combined with hyperphagia for standard chow, may reflect differential programming of homeostatic versus hedonic feeding circuits. Chow consumption is primarily driven by homeostatic mechanisms regulated by hypothalamic circuits, whereas sucrose-seeking reflects hedonic/reward-driven behavior mediated by the mesolimbic dopaminergic system (Russo and Nestler, 2013; Singh, 2014).

Consistent with our findings, in 2014 Treesukosol demonstrated that female offspring from HDF mothers presented reduced appetitive motivation for sucrose (Treesukosol et al., 2026). In contrast, several studies reported higher motivation or preference for palatable food in offspring from mothers fed with HF (Bayol et al., 2007; Vucetic et al., 2010; Ong and Muhlhausler, 2011; Contu et al., 2022). Importantly, Ong and colleagues, differentiated that the preferred macronutrient of offspring from mothers fed with cafeteria diet was fat, but not carbohydrates (Ong and Muhlhausler, 2011). In spite of contradictory observations in behavior, researchers agree that this may reflect a dysregulation of the mesolimbic reward system, which is known to be sensitive to perinatal nutritional insults (Vucetic et al., 2010; Gugusheff et al., 2013; Camacho et al., 2017; Glendining et al., 2018; Contu et al., 2022). These behavior discrepancies highlight that perinatal nutritional programming of motivational circuits is complex and may depend on factors such as the age at testing, the specific reward being measured (sucrose vs. fat), and whether the behavioral task measures appetitive (seeking) versus consummatory (intake) components (da Silva et al., 2013; Paradis et al., 2017; Zeltser, 2018).

The present results of anxiety-like behavior in both behavioral tests (MOFT and L/D-BT) showed that female offspring from-HF-Pa were the most affected, showing reduced anxiety-like behavior and obtaining consistent 4 of 6 parameter evaluated (M-HF-Pa, F-HF-Mo and F-HF-Fa obtained only 1). These findings indicate that combined parental HF exposure exerts a sex-specific anxiolytic-like effect, with females being specifically affected. Similar results were found by Sasaki et al., who demonstrated that maternal HF exposition produced decreased anxiety behavior in offspring (both sexes), an effect explained by alterations in the expression of the glucocorticoid receptor (Sasaki et al., 2014). Other studies have shown opposite results, finding an anxiety phenotype in offspring (males and females) from mothers fed with HF (Glendining et al., 2018; Winther et al., 2018) or offspring with parental HF (Oliveira et al., 2025). A study evaluating the influence of paternal HF also found reduced anxiety-like behavior in female offspring when they were fed a control diet (Freire et al., 2024). Further studies are necessary to determine how anxiety-like behavior is affected by parental diets.

Our behavioral data further indicates that metabolic reprogramming is accompanied by significant alterations in anxiety and food motivation. When examining sexual dimorphism across the findings, it becomes evident that female offspring were more affected than their male counterparts. In addition, the additive effect observed in the HF-Pa groups in anxiety-like behavior and in food-seeking behavior, aligns with emerging evidence that combined maternal and paternal nutritional state influences offspring neurodevelopment, which is reflected in behavior, highlighting the importance of considering sex as a critical biological variable when evaluating the intergenerational effects of parental nutrition.

## 5 Conclusion

This study demonstrates that parental consumption of a high-fat diet acts as an intergenerational risk factor, with the most marked metabolic and behavioral deficits manifesting in offspring when both parents are exposed. The observed phenotype, characterized by hyperphagia, weight gain, diminished anxiety, and reduced motivation for food, suggests a fundamental reprogramming of neurobehavioral circuits governing energy balance and reward. As posited by the DOHaD hypothesis, these developmental alterations produce a co-occurring metabolic and behavioral phenotype (Barker, 2007). Reduce anxiety may reflect a altered reward system reinforcing food intake (Vucetic et al., 2010). Our findings suggest that intergenerational HFD effect extend beyond metabolism to reprogram behavioral regulation. These findings have important translational implications: the prevalence of high-fat diets in modern societies underscores the urgent need for public health interventions targeting parental nutrition, as poor nutrition may affect neurobehavioral outcomes in offspring.

## 6 Limitations

Several limitations should be acknowledged. First, the maternal HF effects observed cannot be attributed solely to preconception exposure, as dams remained on the HF diet during lactation. Both prenatal (gestational) and postnatal (lactational) exposure contributed to the observed offspring phenotypes. Future studies using cross-fostering designs should dissociate gestational from lactational effects to determine the relative contributions of each period. Second, while we observed behavioral changes, we did not directly assess the underlying mechanisms. Future investigations should examine epigenetic modifications (DNA methylation, histone modifications), gene expression changes in reward and stress pathways (e.g., dopamine receptor genes, glucocorticoid receptor, orexigenic/anorexigenic signaling), and neuroanatomical alterations in mesolimbic circuitry. Third, we did not assess F0 behavioral phenotypes; future research should examine whether behavioral changes in parents contribute to offspring outcomes through both genetic and behavioral inheritance mechanisms. Fourth, we evaluated behavior at a single developmental time point (PN40-PN62); longitudinal studies across different developmental stages (juvenile, adolescent, adult) would provide a more comprehensive understanding of the trajectories and persistence of these effects. Fifth, while we observed sex differences in adipose tissue expansion, we did not directly assess adipocyte morphology, adipose tissue inflammation, lipolytic activity, or lipid turnover. Consequently, we cannot determine whether the increased adiposity observed in females reflects healthier adaptive adipose tissue or earlier onset of adipose tissue dysfunction. Future studies incorporating these measures are needed to fully characterize the metabolic consequences of parental HFD exposure in a sex-dependent manner. Finally, investigation of the F2 generation would help determine whether these effects are transgenerational (persisting in germline-unexposed progeny) versus intergenerational in nature.

## Data Availability

The raw data supporting the conclusions of this article will be made available by the authors, without undue reservation.

## Supplementary material

Supplementary Figure 1 F0. Percentage of reproductive success from F0 males **(A)** and females **(B)** fed with Chow Diet **(C)** or High Fat Diet (HF). Number of pups obtained per litter from male rats **(C)** or female rats **(D)** fed with C or HF. Average of body weight of each litter from male rats **(E,G)** or female rats **(F,H)** fed with C or HFD at birth or weaning period. Data are shown as percentage or the mean ± SEM.
