# Supplementary material for: Sex-dependent phenotypes of anxiety and palatable food-seeking in offspring born from rats exposed to a high-fat diet
Source: Front Psychol. 2026 Aug 26;17:1898577. doi: 10.3389/fpsyg.2026.1898577 (PMC13553926; doi:10.3389/fpsyg.2026.1898577)
Supplement: Supplementary file 1 [file Table_1.docx]

**Supplementary Table 1**: Criteria for Food-seeking behavior and Anxiety-like behavior. (n=number of behaviors; s= seconds; %=percentage; ml=milliliters)

| **Criteria for Food-seeking behavior** | | |
| --- | --- | --- |
| **Modified Open Field Test (MOFT)** | High Motivation | Low Motivation |
| Entries into the pellet zone (n) | + | - |
| Latency to interact with the pellet (s) | - | + |
| Interaction time with the pellet (s) | + | - |
| **Light/Dark Box Test (L/D-BT)** | High Motivation | Low Motivation |
| Preference for light compartment (%) | + | - |
| Latency to first drink from the sucrose bottle(s) | - | + |
| Interaction time with the sucrose bottle (s) | + | - |
| Total sucrose intake (ml) | + | - |
| **Criteria for Anxiety-like behavior** | | |
| **Modified Open Field Test (MOFT)** | High Anxiety | Low Anxiety |
| Ambulation (number of visited squares; n) | + | - |
| Entries into the center zone (9 squares; n) | - | + |
| Time spent grooming (s) | + | - |
| Number of fecal boli (n) | + | - |
| **Light/Dark Box Test (L/D-BT)** | High Anxiety | Low Anxiety |
| Transitions between compartments (n) | + | - |
| Latency to enter the dark compartment (s) | - | + |
