# Supplementary material for: Sex-dependent phenotypes of anxiety and palatable food-seeking in offspring born from rats exposed to a high-fat diet
Source: Front Psychol. 2026 Aug 26;17:1898577. doi: 10.3389/fpsyg.2026.1898577 (PMC13553926; doi:10.3389/fpsyg.2026.1898577)
Supplement: Supplementary file 2 [file Table_2.docx]

**Supplementary Table 2.** Percentage of Male (M) and Female (F) subjects that exhibited each motivated behaviors in the Modified Open Field Test (MOFT).

| **MOFT** | | % of subjects | |
| --- | --- | --- | --- |
| Group | Sex | Interaction with pellet | Grooming |
| C-Pa | M | 50 | 100 |
|  | F | 62.5 | 100 |
| HF-Fa | M | **91.6** | 100 |
|  | F | **91.6** | 100 |
| HF-Mo | M | **91.6** | 100 |
|  | F | **91.6** | 100 |
| HF-Pa | M | **75** | **91.6** |
|  | F | **100** | **91.6** |
