# Supplementary material for: Sex-dependent phenotypes of anxiety and palatable food-seeking in offspring born from rats exposed to a high-fat diet
Source: Front Psychol. 2026 Aug 26;17:1898577. doi: 10.3389/fpsyg.2026.1898577 (PMC13553926; doi:10.3389/fpsyg.2026.1898577)
Supplement: Supplementary file 3 [file Table_3.docx]

**Supplementary Table 3.** Percentage of male (M) and female (F) subjects that exhibited each motivational behaviors in the Light/Dark Box Test (L/D-BT).

| L/D-BT | | % of subjects | |
| --- | --- | --- | --- |
| Group | Sex | Drinking bottle | Interaction with bottle |
| C-Pa | M | 100 | 100 |
|  | F | 87.5 | 87.5 |
| HF-Fa | M | **83.3** | **91.6** |
|  | F | **91.6** | **91.6** |
| HF-Mo | M | **100** | **100** |
|  | F | **100** | **100** |
| HF-Pa | M | **83.3** | **91.6** |
|  | F | **66.6** | 83.3 |
